# Accuracy, Stability, and Corrective Behavior in a Visuomotor Tracking Task: A Preliminary Study

**DOI:** 10.1371/journal.pone.0038537

**Published:** 2012-06-04

**Authors:** Young U. Ryu, John J. Buchanan

**Affiliations:** 1 Department of Physical Therapy, Catholic University of Daegu, Gyeongsan, South Korea; 2 Department of Health & Kinesiology, Texas A & M University, College Station, Texas, United States of America; The University of Western Ontario, Canada

## Abstract

Visuomotor tracking tasks have been used to elucidate the underlying mechanisms that allow for the coordination of a movement to an environmental event. The main purpose of the present study was to examine the relationship between accuracy and stability of tracking performance and the amount of corrective movements that emerge for various coordination patterns in a unimanual visuomotor tracking task. Participants (N = 6) produced rhythmic elbow flexion–extension motions and were required to track an external sinusoidal signal at five different relative phases, 0°, 45°, 90°, 135°, and 180°. Differential accuracy and stability were found among the five tracking patterns with the 0° relative phase pattern being the most accurate and stable pattern. Corrective movements were correlated with changes in accuracy only for the 0° relative phase pattern, with more corrections emerging for less accurate performance. The amount of corrective movements decreased as the stability of tracking performance increased for the 0°, 45°, and 135° patterns. For the 90° and 180° tracking patterns, the amount of corrective movements was not correlated with pattern accuracy or pattern stability. The results demonstrate that corrective behaviors are an important motor process in maintaining the stability of stable perception-action coordination patterns, while offering little benefit for unstable perception-action patterns.

## Introduction

Humans have the ability to synchronize limb motions to changing environmental conditions under both expected and unexpected conditions. This ability to adapt limb motions to changing environmental conditions requires the neuro-motor system to make corrections in a limb's trajectory while maintaining accuracy and stability. Visuomotor tracking tasks have been used to elucidate the underlying mechanisms that support corrective adjustments when tracking a moving object based on visual information [1], [2], [3], [4]. In visuomotor tasks examining corrective responses, participants typically track a rhythmic external signal in an in-phase manner characterized by 0° phase lag between the external signal and a limb. Corrective behavior in such visuomotor tracking tasks may be characterized by directional changes in the velocity profile of the tracking limb's trajectory and measured by an increase in power (referred to as intermittency) in a frequency band higher than the target signal's frequency [1], [2]. Several studies have demonstrated that the degree of intermittency is dependent on the utilization of feedback information [2], [3]. For example, intermittency (fewer corrections) in visuomotor tracking is reduced as the predictability of target motion increases, when online visual feedback of the actor's response is not presented [2], when target frequency or velocity is increased [1], and with an increase in the amount of practice [4].

The visuomotor tracking paradigm has also been used to investigate pattern accuracy and pattern stability within the perception-action cycle [5], [6], [7]. In this paradigm, participants are required to rhythmically track an external signal at various phase relations (not just 0° in-phase). Such studies have revealed that in-phase tracking is more accurate and stable than anti-phase tracking (180° phase lag) [5], [6], [7], [8], [9], and that an intermediate tracking pattern (90° phase lag) is less accurate and stable compared to in- and anti-phase tracking [5], [10]. This difference in accuracy and stability found between different visuomotor coordination patterns can be understood in terms of the dynamical process of entrainment between two oscillators (environmental event and actor) that are coupled via visual information [1], [7], [11]. That is, the anti-phase tracking pattern is less stable because the coupling strength between the external to-be-tracked target signal and the actor's limb motion is less in comparison to in-phase tracking [11].

**Figure 1:**
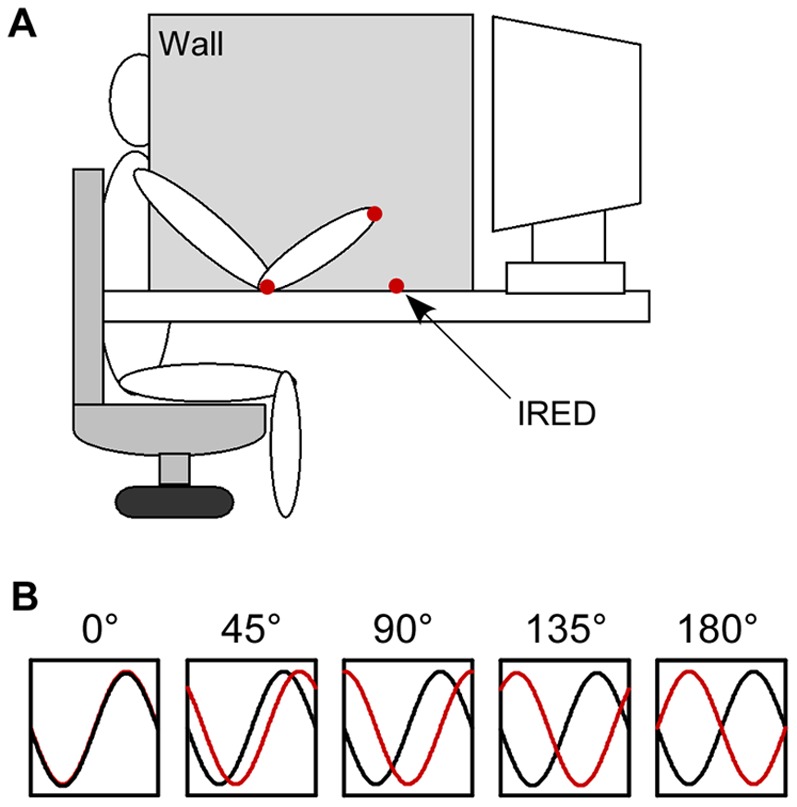
Experimental setup and the required target patterns. In the required patterns (B), the black curve represents the external signal and the red curve represents the required signal that a participant is supposed to produce.

Even though the visuomotor tracking paradigm has been used to explore both corrective processes and performance accuracy linked to tracking stability, it is still unknown if the differential accuracy and stability of various tracking patterns is linked to differential levels of intermittency. In order to examine this question, we adapted a visuomotor tracking experiment previously used by Ryu and Buchanan (2009). Participants tracked a sinusoidal wave with elbow flexion-extension motion at five relative phase patterns (Figure 1). The main purpose of the present study was to examine the relation between tracking accuracy and stability and the level of corrective behavior in the form of intermittency. Are some visuoumotor tracking patterns less stable due to the inability of individuals to perceive that corrections are required or are they less stable because of more corrections being made? Research has shown that stable coordination patterns such as in-phase and anti-phase are perceived as more coordinated than less stable coordination patterns such as 90° [12], [13]. The perceptual evaluation findings mirror the production findings, with in-phase and anti-phase produced very stably without the need for extensive practice [14], [15], [16], whereas 90° is unstable and can require extensive practice to produce [17], [18], [19]. Bingham and colleagues assume that if a variable such as relative phase is to be controlled it must also be perceptible [12]. What is examined with this study is the reverse of the above assumption, if a variable can be perceived, can that perception help with control if what is to be controlled (i.e., a relative phase pattern) is unstable. Based on the above assumption, it is predicted that visuomotor tracking patterns that are more stable and perceived as more coordinated will require less perceptual costs (decreased effort in processing feedback) and be associated with less intermittency. Less stable visuomotor tracking patterns (e.g., a 90° relative phase pattern), however, will require more perceptual cost to produce and result in an increase in intermittency compared to stable visuomotor tracking patterns.

## Materials and Methods

### Subjects

The protocol and consent form for this study were approved by the Institutional Review Board of Texas A&M University. Each participant signed a written consent form prior to participating. Six participants (3 males and 3 females, mean age  = 22.4 yrs) volunteered to participated in the study and all six participants were self-reported right-handers with normal or corrected-to-normal vision.

### Apparatus and protocol

Participants sat on a height-adjustable chair with their right elbow comfortably placed supine on a table. The participant's wrist was secured with a wrist orthosis in parallel with the longitudinal axis of the forearm. A barrier (0.84 m×0.48 m) was mounted on a table between the participants' head and arm. The participant could not see their forearm motions. A computer monitor was placed 0.76 meters in front of the participant and used to display the external sinusoidal signal and trace representing the participant's forearm motion (Figure 1A). The sinusoidal signal was produced with a function generator (Tektronix® AFG320, SONY) with the oscillation frequency set at 0.8 Hz with 8 cycles in a trial. The oscillation frequency of 0.8 Hz was chosen because previous studies have shown that differential stability in tracking pattern can be observed at this rate (e.g., 0.67 Hz in [9], 1 Hz in [8]). A motion capture camera system (OPTOTRAK® 3020, Northern Digital) recorded the position of three infrared light emitting diodes (IREDs). The IREDs were mounted on a dowel held in the hand, the lateral epicondyle of the elbow, and the table corresponding to the dowel's height when the participant's hand was extended to maximum extension along the table (Figure 1A). The participant's elbow angular motion was represented by the change in the angle θ defined by the dowel's position with regard to the table mounted IRED. Participants were asked to flex and extend their right elbow in order to produce a sinusoidal signal that would track the external sinusoidal signal at a specific offset. The sinusoidal tracking signal and the participant's motion signal were superimposed in a single window display (14 cm long ×4.5 cm high) presenting two peak-to-peak cycles of the external signal at a time.

Participants were given 4 familiarization trials with the flexion-extension motion of elbow displayed on the computer screen. For these trials, the oscillation frequency was set at 0.5 Hz with a total of 8 cycles in a trial. This oscillation frequency was slower than the one used in the experimental sessions (0.8 Hz) to reduce the practice effect on tracking before the experiment. The experimental trials were conducted right after the familiarization trials, and the experimental trials consisted of 3 trials each for 5 different relative phase patterns: 0°, 45°, 90°, 135°, and 180° (Figure 1B). The relative phase patterns were defined between the external sinusoid signal and the online visual feedback of the elbow's motion. Before performing each relative phase pattern, the required relative phase pattern was simulated. In the simulation, the external signal was presented as a black sinusoid and a simulated signal of the participant's elbow motion was represented by a red trace. In the simulation trials, the participant's simulated signal lagged the external signal in the 45°, 90°, and 135° relative phase pattern. Participants were asked to reproduce the simulated pattern when they performed. After watching the simulation of a given relative phase pattern, participants immediately performed 3 consecutive trials for that relative phase pattern. The presentation of the five relative phase patterns was randomized across participants. Studies that have examined bimanual learning of relative phase patterns such as 90° often scan across many relative phase patterns in an increasing and decreasing order from 0° (in-phase) to 180° (anti-phase) in order to test for attraction to the 90° relative phase pattern [17], [20]. The intention of this study was not to examine attraction to a novel pattern following training, but was instead to determine if differences in tracking accuracy and stability were linked to corrective actions. For this reason, the experimental design was based on the more classical approach of presenting the five relative phase patterns in a randomized order instead of ascending and/or descending scaling order.

### Data Analysis

Prior to data analysis, a dual-pass Butterworth Filter was applied to the 3D IRED trajectories with a cutoff frequency of 10 Hz. The filtered data were used to compute the elbow joint angle. The first cycle of motion was dropped and considered as an adaptation phase so that 7 cycles were analyzed from each trial. A continuous relative phase was computed to characterize the spatiotemporal tracking relationship between the external signal and the elbow angle. For both the tracking signal (*θ*_external_) and elbow angle (*θ*_elbow_), individual phase angles were computed for every sampled point *i* in a trial, *θ_i_*  =  tan^−1^[(*dx_i_* /*dt*)/*x_i_*], with *x_i_* the normalized position and *dx_i_*/*dt* the normalized instantaneous velocity. For each sampled point *i* in a trial, the continuous relative phase was computed as *φ_i_*  =  *θ*_external_ − *θ*_elbow_. Mean resultant vectors were computed from the observed unit vectors (*x*, *y*) of the*φ_i_* values [21]. The mean resultant vectors were used to compute a mean phase angle (*φ_obs_*) and a magnitude of circular variance. A value of *φ_obs_*  = 0° represented in-phase tracking and a value of *θ_obs_*  = 180° represented anti-phase tracking. There were five required relative phase values, *φ_req_*  = 0°, −45°, −90°, −135°, and 180°. An absolute phase error (*φ_AE_*  =  | *φ_req_* − *φ_obs_*|) was computed and used to evaluate tracking accuracy. Circular variance was computed to characterize the variability in the visuomotor tracking patterns. The circular variance measure falls within the range of 0 to 1 with one representing perfect uniformity. In order to submit the circular variance data to inferential tests based on standard normal theory [22], the circular variance score was transformed to the range 0 to ∞ as follows, s_0_  =  −2log_n_(1 − S_0_)^0.5^. S_0_ denotes the measure of circular variance on the interval (0, 1) with s_0_ the transformed circular variance (TCV) measure that was submitted to statistical analysis. This TCV measure was treated as an estimate of pattern stability in tracking performance. The transformation applied to the circular variance scores results in smaller TCV values indicating more variability or less stable tracking performance.

**Figure 2:**
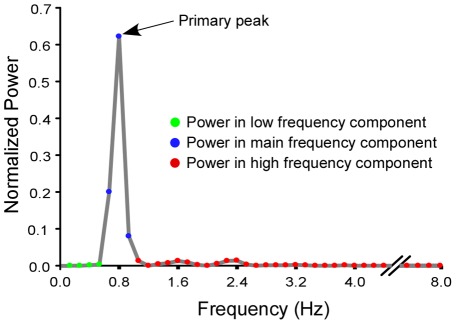
An example power spectrum based from an FFT of the elbow's velocity signal. This example is from a single trial with a required relative phase of 0°. The three frequency regions are highlighted. The tracking frequency was 0.8 Hz. The high frequency component, representing the measure of intermittency, is highlighted with the red circles.

A discrete Fourier transform was applied to the angular velocity time series with a Hanning window, and the resolution of power spectrum was 0.133 Hz. The resulting power spectrum was analyzed to determine the level of intermittency in each participant's performance. The power spectrum provided information about the distribution of power across the frequency components of a signal. The power spectrum also provided the frequency of the primary peak which was defined as the frequency at the largest power in the spectrum (Figure 2). The distribution of the power spectrum for each trial was normalized by the total power. From the normalized power spectrum three frequency components (low, main, and high) were defined (Figure 2). The main frequency component of the distribution was defined as ±0.133 Hz around the frequency of the primary peak. The high frequency component was defined as those frequencies above the main frequency component and the low frequency component was defined as the frequencies below the main frequency component. The high frequency component is the measure of intermittency with a larger proportion in the higher frequency range indicating more intermittent (corrective) performance during tracking. The low frequency component was not used for statistical analysis since it consisted of less than 1% of the total power distribution across all trials and was beyond the purpose of the present study.

Dependent variables were the absolute phase error (*φ_AE_*), TCV, mean power frequency, and the percentage of power in the main and high frequency components. Two statistical analyses were used. A repeated measures ANOVA was performed on each dependent variable with five levels of Pattern (0°, 45°, 90°, 135°, 180°). Tukey's HSD test was used to reveal differences between patterns when a main effect was present. Partial eta squared values (η_p_^2^) were computed and have been reported with every significant F value. Bivariate correlations between absolute phase error and the percentage of power in the high frequency component, and between TCV values and the percentage of the power in the high frequency component were performed on the data for each of the five relative phase patterns and Pearson's correlation coefficient (*r*) is reported. The significance level α was set at 0.05 for the test of statistical significance.

## Results

**Figure 3:**
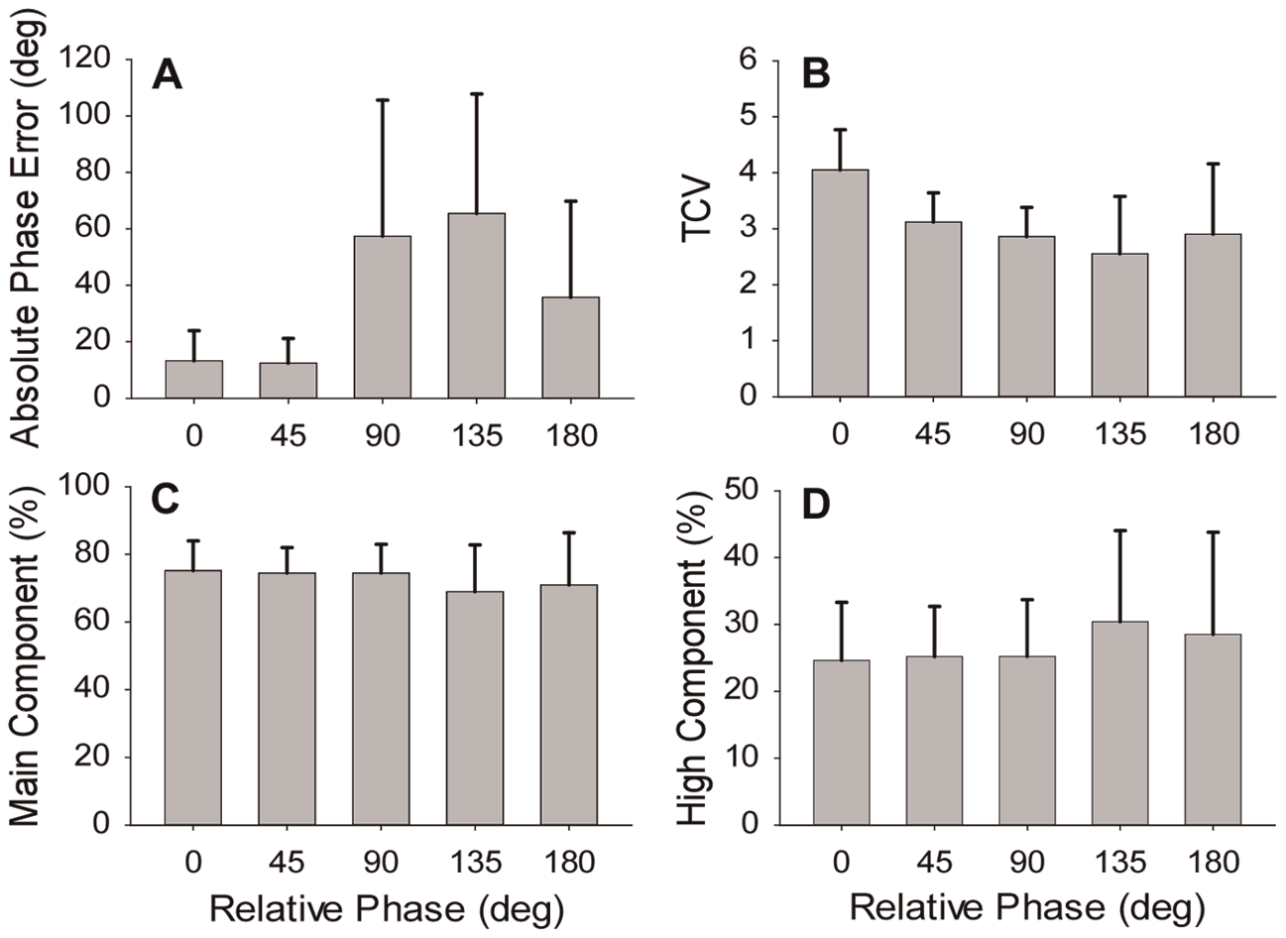
Mean absolute phase errors, TCV values, and percentages of main and high frequency components as a function of required target pattern. Differential accuracy and stability were found among the five tracking patterns with the 0° relative phase pattern being the most accurate and stable pattern (A and B). Differential intermittency among the five patterns was not found (D). The error bars represent the standard deviation of the between subject variability.

A main effect of pattern was found in the *φ_AE_* data (*F*_(4,20)_ = 4.36, p<.05, *η_p_* ^2^ = .47) and post-hoc tests revealed that the 0° and 45° target patterns were produced with significantly less error than the other three patterns (Figure 3A). The analysis of the TCV values revealed a main effect of Pattern (*F*_(4,20)_ = 6.93, p<.01, *η_p_* ^2^ = .69) and post-hoc tests showed that the 0° phase pattern more stable (largest TCV value) than the other four patterns and that the 135° phase pattern was less stable (lowest TCV value) than the other four patterns (Figure 3B), with no differences between 45°, 90° and 180° patterns. The power spectrum analysis showed that the primary peak was located at the target frequency (Mean  = 0.801 Hz, Std. Dev.  = 0.004) and that peak frequency was not significantly affected by visuomotor tracking pattern (p>0.5). No significant main effects or interactions were found in the analysis of the percentage of power in the main frequency component. This was expected because the required tracking frequency was the same regardless of the required tracking pattern. The analysis of the percentage of power in the high frequency component did not reveal a main effect of pattern (Figure 3D). This result does not satisfy the prediction of finding differential intermittency as a function of differences in tracking accuracy and stability.

**Figure 4:**
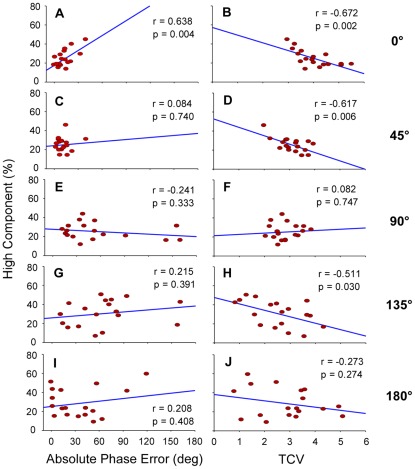
Results of correlation analyses. Correlations between absolute phase error and high component percentage (A, C, E, G, and I), and between TCV and high component percentage (B, D, F, H, and J) are plotted as a function of required relative phase: 0°, 45°, 90°, 135° and 180°. Corrective movements were correlated with changes in accuracy only for the 0° target pattern (A). The amount of corrective movements decreased as the stability of tracking performance increased for the 0°, 45°, and 135° patterns (B, D, and H). For the 90° and 180° target patterns, the amount of corrective movements was not correlated with pattern accuracy (E and I) or pattern stability (F and J). Pearson's correlation coefficients (r) and p-values are presented. Blue lines represent correlation lines.

The bivariate correlation analysis between absolute phase error and the percentage of power in the high frequency component for the 0° tracking pattern revealed that intermittency was significantly correlated with pattern accuracy (Figure 4A). The positive correlation suggests that intermittency increases as tracking accuracy decreases. The correlation between phase error and the high frequency component of the power was not significance for the other four patterns (Figure 4C, E, G, and I). Significant correlations between the percentage of power in the high frequency component and the TCV values were found for the 0°, 45° and 135° patterns (Figure 4B, D, and H). These significant negative correlations suggest that intermittency increases when the stability of tracking performance decreases. The correlation analyses between TCV values and the percentage of power in the high frequency component for the 90° and 180° tracking patterns were not statistically significant (Figure 4F and J).

## Discussion

In the present study, unimanual elbow flexion-extension coordination with an external sinusoidal signal was performed across five relative phases at 0°, 45°, 90°, 135°, and 180°. The five visuomotor tracking patterns were assessed in terms of accuracy, variability, and the amount of corrective behavior as indexed by the high frequency component in the elbow angle velocity profile. The primary prediction of the current study was that intermittency would increase as performance error increased and performance stability decreased, thereby reflecting more corrective actions when tracking performance was poor. The primary prediction was not borne out by the analysis of the high frequency component. The correlation analysis revealed that the intermittency measure was more correlated with pattern stability (TCV measure) than pattern accuracy.

Overall, 0° in-phase was the visuomotor tracking pattern produced with the least amount of error and was the most stable pattern produced. The relationship between intermittency, accuracy, and stability was strongest for this pattern, with the power of the high frequency component increasing as error and variability in this pattern increased. Research on perceptual discrimination processes has demonstrated that different levels of variability may be perceived when two dots move in-phase with each other [12], [13]. This ability to discriminate variability levels is weaker for dots moving at 180° anti-phase and is non-existent for dots moving at relative phases from 30° to 150° (in 30° steps). The correlations between intermittency and phase error and variability for the in-phase pattern are consistent with the perceptual evaluation studies of Bingham and colleagues and strongly suggest that if something is to be controlled aspects (e.g., variability) of it must be perceivable.

A strong correlation was also observed between coordination variability and the high frequency component for the 45° and 135° patterns. For the 45° relative phase pattern, mean error was small and variability was only second to 0° in-phase. This indicates that corrective actions were utilized to some extent, but maybe not as successfully in comparison to the 0° in-phase pattern. The 135° relative phase pattern was characterized by a large group error, and in fact many trials for this condition were showing an attraction to 0° and 45°. Thus, the significant correlation between stability and the high frequency component for the 135° pattern emerged because of an attraction to more stable patterns when attempting this unstable pattern. This sensitivity to variability when attempting the 45° and 135° pattern is distinct when placed in the context of the work on perceptual evaluation where variability differences in a perceptual display were only identified for the 0° and 180° [12], [13]. The tasks used in the perceptual evaluation work did not have a production component that was producing half of the perceptual pattern as in the current task. This suggests that when perception and production are linked through the task that the perceptual system may be more sensitive of underlying motor processes. This is exactly what should be expected if learning is the process whereby perceptually defined environmental information transforms an unstable coordination pattern into a stable coordination patterns [17], [23], [24], [25].

For the 90° and 180° tracking patterns, however, a relationship between pattern accuracy and stability and intermittency was not observed. Perceptually, a 90° relative phase pattern represented as moving dots on a computer screen is judged as less coordinated compared to 0° and 180° which are judged as the most coordinated [12]. Variability differences in a 90° relative phase pattern are also not perceptually distinguishable [12], [13]. When viewed in light of the perceptual evaluation work, the finding that corrective actions were not correlated with performance accuracy or stability when attempting 90° is not so surprising. This implies that errors in performance only carry meaning when an action is stable and can be perceived as stable. Under this condition, errors carry meaning that allow for corrective action to occur such that accuracy and stability are maintained. For some coordination patterns, performance instability may not allow for consistent error detection and therefore corrective actions are not emerging to improve accuracy and increase stability outside of a learning enhanced context [24]. The results with regard to the 180° pattern were unexpected, especially when placed in the context of the perceptual evaluation work that has shown a sensitivity of the perceptual system to variability in this pattern. Again, this may be related to the difference in the type of tasks, with the current perceptual pattern constructed from both a motor and perception only component. The alternating peak and valley events for this pattern may have made it easier to produce, but the greater distance between the peaks and valleys of the two signals may have made it harder determine variability and execute corrective actions consistently [26].

In the statistical analysis of the high frequency components a difference in intermittency among the five coordination patterns as predicted was not found. As outlined above, the correlation analysis suggests that intermittency was playing a role based on the variability data, but not in a manner consistent with the perceptual evaluation work for all five patterns. One plausible explanation for the present findings is that the external signal and limb motion signal were presented as overlapping sine waves [10] and not as two dots as in the perceptual evaluation work [12], [13]. Another issue that may have contributed to the current findings was the random presentation of the relative phase patterns and relatively short trial lengths of 8 secs. The random presentation requires participants to expend effort dealing with the switch between patterns and this in combination with short trials may have resulted in large transients (possibly longer than the 1 cycle that was dropped). The current findings show that the measure of intermittency derived from the frequency analysis is a sensible way to characterize corrective behavior in a perception-action system. Future research needs to utilize this measure with studies that examine how different visual displays (sine waves versus cursors) may influence perceptual evaluations and corrective behavior. Moreover, future research needs to more thoroughly explore the learning aspect of corrective behavior through extensive training of various relative phase patterns utilizing block designs and longer trial lengths.

## References

[1] Russell DM, Sternad D (2001). Sinusoidal visuomotor tracking: intermittent servo-control or coupled oscillations?. Journal of Motor Behavior.

[2] Miall RC, Weir DJ, Stein JF (1993). Intermittency in human manual tracking tasks.. Journal of Motor Behavior.

[3] Craik KJW (1947). Theory of the human operator in control systems I. The operator as an engineering system.. British Journal of Psychology-General Section.

[4] Noble M, Fitts PM, Warren CE (1955). The frequency response of skilled subjects in a pursuit tracking task.. Journal of Experimental Psychology.

[5] Wilson AD, Collins DR, Bingham GP (2005). Perceptual coupling in rhythmic movement coordination: stable perception leads to stable action.. Experimental Brain Research.

[6] Peper CE, Beek PJ (1998). Are frequency-induced transitions in rhythmic coordination mediated by a drop in amplitude.. Biological Cybernetics.

[7] Wimmers RH, Beek PJ, Van Wieringen PCW (1992). Phase transitions in rhythmic tracking movements: A case of unilateral coupling.. Human Movement Science.

[8] Buekers MJ, Bogaerts HP, Swinnen SP, Helsen WF (2000). The synchronization of human arm movements to external events.. Neuroscience Letters.

[9] Ceux T, Buekers MJ, Montagne G (2003). The effects of enhanced visual feedback on human synchronization.. Neuroscience Letters.

[10] Ryu YU, Buchanan JJ (2009). Learning an environment-actor coordination skill: visuomotor transformation and coherency of perceptual structure.. Experimental Brain Research.

[11] Snapp-Childs W, Wilson AD, Bingham GP (2011). The stability of rhythmic movement coordination depends on relative speed: the Bingham model supported.. Experimental Brain Research.

[12] Bingham GP, Schmidt RC, Zaal FTJM (1999). Visual perception of the relative phasing of human limb movements.. Perception and Psychophysics.

[13] Zaal FTJM, Bingham GP, Schmidt RC (2000). Visual perception of mean relative phase and phase variability.. Journal of Experimental Psychology: Human Perception and Performance.

[14] Kelso JAS (1984). Phase transitions and critical behavior in human bimanual coordination.. American Journal of Physiology [Reg Integ Comp].

[15] Kelso JAS, Scholz JAS, Schöner G (1986). Nonequilibrium phase transitions in coordinated biological motion: critical fluctuations.. Physics Letters A.

[16] Schöner G, Haken H, Kelso JAS (1986). A stochastic theory of phase transitions in human hand movement.. Biological Cybernetics.

[17] Zanone PG, Kelso JAS (1992). The evolution of behavioral attractors with learning: nonequilibrium phase transitions.. Journal of Experimental Psychology: Human Perception and Performance.

[18] Zanone PG, Kelso JAS (1997). Coordination dynamics of learning and transfer: collective and component levels.. Journal of Experimental Psychology: Human Perception and Performance.

[19] Lee TD, Swinnen SP, Verschueren S (1995). Relative phase alterations during bimanual skill acquisition.. Journal of Motor Behavior.

[20] Tallet J, Kostrubiec V, Zanone PG (2008). The role of stability in the dynamics of learning, memorizing, and forgetting new coordination patterns.. Journal of Motor Behavior.

[21] Burgess-Limerick RJ, Abernethy B, Neal RJ (1991). Note: A statistical problem in testing invariance of movement using the phase plane model.. Journal of Motor Behavior.

[22] Mardia KV (1972). Fundamentals of experimental design..

[23] Wilson AD, Snapp-Childs W, Bingham GP (2010a). Perceptual Learning Immediately Yields New Stable Motor Coordination.. Journal of Experimental Psychology-Human Perception and Performance.

[24] Wilson AD, Snapp-Childs W, Coats R, Bingham GP (2010b). Learning a coordinated rhythmic movement with task-appropriate coordination feedback.. Experimental Brain Research.

[25] Schöner G, Zanone PG, Kelso JAS (1992). Learning as change of coordination dynamics: theory and experiment.. Journal of Motor Behavior.

[26] Wickens CD, Carswell CM (1995). The proximity compatibility principle: its psychological foundation and relevance to display design.. Human Factors.

